# Clostridium *sordelli* causing malignant edema in a trauma patient: a case report and review of literature

**DOI:** 10.11604/pamj.2018.30.118.14156

**Published:** 2018-06-12

**Authors:** Sanjiv Fitz-Morris Gray, Beatrice Esther Dieudonne

**Affiliations:** 1 University of Central Florida, Osceola Regional Medical Center, Orlando, Florida, USA

**Keywords:** Sordelli, malignant edema, clostridium

## Abstract

Clostridium *sordelli* infection is a rare but potentially lethal infection associated with abortions, injury, contaminated wounds, and illicit drug use. It causes a recognizable syndrome with marked leukocytosis. Rapid diagnosis and aggressive sepsis management is required for optimal outcome. We report a case in a trauma patient with delayed presentation after sustaining facial trauma with soil contamination. The critical care management is summarized and a review of the literature.

## Introduction

We are reporting a case of malignant edema secondary to Clostridium *sordelli* infection in a trauma patient after blunt trauma to the face. C. *sordelli* is an anaerobic, gram- positive, spore forming rod with peritrichous flagella. Virulent strains can cause lethal infections such as enteritis and enterotoxaemia in sheep and cattle and myonecrosis and gangrene in humans. Most of our knowledge of this pathogen is derived from case reports and small case series. Increased awareness of this potentially lethal pathogen can aid acute care surgeons with rapid diagnosis when encountering similar presentations.

## Patient and observation

**Figure 1:**
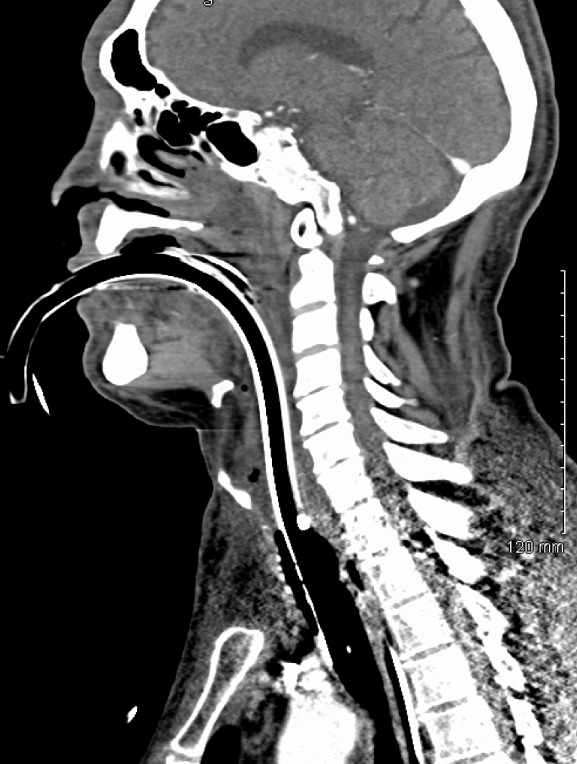
Extensive neck and upper torso edema

**Figure 2:**
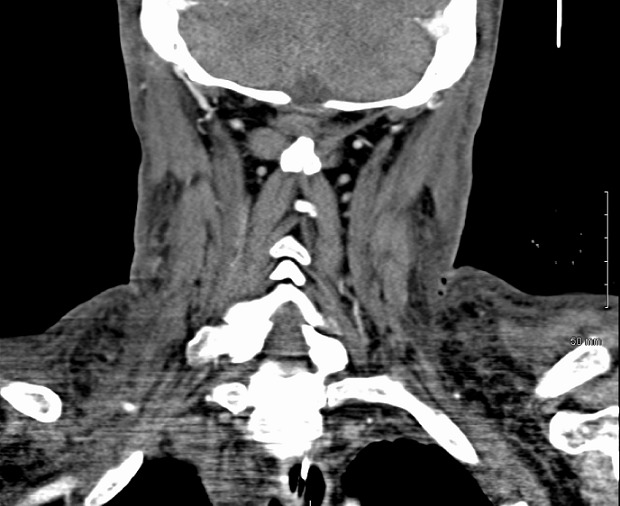
Torso edema with gas

61 years old Caucasian male with past medical history of hepatitis C, cirrhosis, hepatocellular carcinoma, alcohol dependence, chronic obstructive pulmonary disease, and hypertension was transferred to our trauma center from a referring facility. The patient was a pedestrian struck by a car five days earlier and sustained a 1.5 centimeter laceration to his right forehead, which was repaired in the emergency department with interrupted sutures. The patient left the facility against medical advice. The patient returned three days later to the outside facility with complaints of increasing right eye pain. It was recommended that he be admitted for further evaluation by ophthalmology and surgery; however, patient refused further treatment and left against medical advice again. The patient returned a day later with increasing facial edema, right eye pain and difficulty breathing. Computed tomography (CT) scan performed showing subglottic edema, edema in the upper chest, right face and neck concerning for severe cellulitis along with trace retropharyngeal and mediastinal fluid Figure 1, Figure 2. The patient was intubated for airway protection, started on empiric vancomycin, clindamycin and piperacillin/tazobactam and transferred to our tertiary center. On arrival he was noted to have bilateral facial swelling, 1.5 cm wound on the right forehead, ecchymosis to the right cheek and right periorbital region and generalized pitting edema over the entire neck and face extending down to the neck and upper chest. Soft palate and uvula swelling were also noted. The patient was afebrile with a white blood cell count of 26 x 10^9^ per liter (L). On hospital day one the patient underwent a tracheostomy which proved difficult due to the extensive edema. The right forehead wound was debrided with findings of mucopurulent drainage, with 6 cm of necrotic tissue excised deep to the tempo-parietal fascia and submitted for cultures. The wound was found to be tracking inferiorly to the temporoparietal region so a tunneling maneuver was done and a right supraauricular incision made and a Penrose drain brought through for further irrigation. On hospital day two the patient's leukocytosis worsened to 37 x 10^9^ per liter (L), developed oliguria, and the swelling continued to extend down to the upper abdomen. Invasive hemodynamic monitoring was used to monitor central venous pressure, cardiac index and stroke volume variation using Vigileo Flo Trac sensor. Preliminary microbiology cultures grew gram positive cocci and gram negative rods from the wound, admission nasal swab was positive for Methicillin/Oxacillin resistant staphylococcus aureus (MRSA); blood, bronchioalveolar lavage and urine cultures were all negative. On hospital day three the patient's upper torso and facial swelling were tenser with the inability to open his eyes and progression to multisystem organ failure. The wound culture grew yeast in addition to previous cultures and therapy was broaden to caspofungin since he was hemodynamically unstable at the time. On hospital day four cultures from the wound resulted to klebsiella pneumoniae, Beta hemolytic Streptococcus (not group A or B) and yeast (not candida albicans) antibiotics were changed to linezolid, pipercillin/tazobactam and caspofungin. On hospital day five cultures grew out MRSA and Candida parapsilosis and caspofungin was step downed to fluconazole along with the continuation of linezolid and pipercillin/tazobactam. Over the next couple days, the patient improved clinically in his mental status, renal function and upper torso edema. On hospital day seven the wound culture also showed Clostridium *sordelli*, which was sensitive to pipercillin/tazobactam per published studies as it could not be established in vitro for the isolate. The antimicrobial regimen was continued and the patient showed marked improvement of his swelling and was weaned off the ventilator. The patient was eventually transferred to a long term acute care facility on hospital day twelve. The patient received twelve days of appropriate coverage with pipercillin/tazobactam for klebsiella pneumonia and clostridium *sordelli*, MRSA coverage with vancomycin and linezolid, and antifungal therapy with caspofungin and fluconazole. Unfortunately, the patient has not followed up at our facility since discharge. Pathological assessment of the debrided tissue showed focally ulcerated skin with necrosis and marked acute inflammation involving dermis, subcutaneous tissue and skeletal muscle with vascular thrombosis and inflammatory exudate.

## Discussion

Clostridium *sordelli* was originally described by Dr Alfredo Sordelli in South America in 1922 as Bacillus *oedematic sporogenes* due to the shared pathogenicity of B. *oedematiens* and the morphologic and cultural properties of B *sporogenes* [1]. In 1927 it was renamed to clostridium *sordelli* to avoid taxology confusion [2]. C. *sordelli* is commonly found in the soil and in the intestines of animals and rarely in humans. Virulence is secondary to the lethal and hemorrhagic toxins. Aldape et al [3] did a comprehensive review of 45 reported cases of C. sordelli infection between 1927 and 2006. 63% of the infections were in women with 35% associated with normal childbirth, 22% associated with medically induced abortion and 9% associated with spontaneous abortion. The mortality rate was 100%. Most of the infections were polymicrobial leading some to believe infection may be secondary to fecal contamination secondary to vaginal delivery or ascending infection through the cervix as clostridia species is known to colonize the vaginal flora of 4-18% of healthy women. There have been 5 reported mifepristone associated medically induced abortion deaths worldwide all of which occurred in the United States and 80% were in California causing the Food and Drug Administration to issue a public health advisory warning but the exact mechanism is not known [4, 5]. Another 22% (10/45) of the cases were associated with intravenous drug users with a 50% mortality. Other 42% (19/45) occurred after traumatic injuries or non-gynecological surgeries in healthy individuals with a mortality rate of 53%. C. *sordelli*infections have been described in patients with alcoholism, cirrhosis, malignancy and immunosuppression without obvious portal of entry [6]. Of note, all these risk factors were present in the patient we presented. The overall mortality is 69% with patients dying rapidly from multiorgan failure. The clinical features of C. *sordelli* infection includes dizziness, lethargy, tenderness and skin changes at the site of infection. There are six distinct clinical features which have been noted: leukomoid reaction, refractory hypotension, severe tachycardia, profound capillary leak syndrome, hemoconcentration, and lack of fever marking the C. sordelli toxic shock syndrome. The leukomoid reaction is characteristic of the clostridium species with similar reaction seen with clostridium difficile. In majority of the case reports patients with WBC > 75000 died while those with less than 18000 survived. C *sordelli*produces 7 identified exotoxins: lethal toxin, hemorrhagic toxin, hemolysin, neuraminidase, phospholipase C, DNAse, and collagenase. The main virulence has been attributed to lethal toxin and hemorrhagic toxin [7]. These toxins are members of the large clostridial cytotoxin (LCC) family, which also include the C. difficile toxins A and B and C. novyi a-toxin. Clinicians must maintain a high degree of suspicion in patients presenting 2-7 days after an injury, surgical procedure, illicit intravenous drug use, childbirth or medically induced childbirth as the clinical presentation is non-specific. Management with early sepsis management with surgical debridement and commencement of broad spectrum antibiotics is recommended. Antitoxin antibiotics maybe beneficial (i.e, clindamycin or linezolid). Literature review reveals the following sensitivities for C sordelli clindamycin 56%, metronidazole 100%, piperacillin/tazobactam 100%, cefoxitin 47%, and ampicillin/sulbactam 100%. In our case, the septic shock was secondary to a polymicrobial infection with features of the C sordelli toxic shock syndrome. The airway was secured and sepsis management done. Antibiotic sensitivities were followed appropriately along with wound care. The patient survived this highly lethal infection and was discharge to a long-term care facility.

## Conclusion

Clostridium *sordelli* infection in post-traumatic wounds is a rare but potentially lethal infection. A high degree of suspicion is required in patients with a history of trauma with soil contamination presenting with leukomoid reaction and severe sepsis. Broad spectrum antimicrobials are recommended along with the addition of anti-toxin agent and early surgical debridement.

## Competing interests

The authors declare no competing interests.

## References

[1] Louis De Spain Smith, Betsy L, Williams Charles C (1984). The Pathogenic Anaerobic Bacteria.

[2] Hall IC, Scott JP (1927). Bacillus sordelli, a cause of malignant edema in man. J Infect Dis.

[3] Aldape MJ, Bryant AE, Stevens DL (2006). Clostridium sordelli infection: epidemiology: clinical findings and current perspectives on diagnosis and treatment. Clin Infect Dis.

[4] Centers for Disease Control and Prevention US Food and Drug Administration and the National Institute of Allergy and Infectious Diseases. Meeting transcript for Public Workshop on Emerging Clostridial Disease (Atlanta) 11 May 2006.

[5] Fischer M, Bhatnagar J, Guarner J (2005). Fatal toxic shock syndrome associated with clostridium sordellii after medical abortion. N Engl J Med.

[6] Bodey GP, Rodriguez S, Fainstein V, Elting LS (1991). Clostridial bacteraemia in cancer patients: a 12 year experience. Cancer.

[7] Arseculeratne SN, Panabokke RG, Wijesundera S (1969). The toxins responsible for the lesions of Clostridium sordelli gas gangrene. J Med Microbiol.

